# Design and Preparation of Nano-Lignin Peroxidase (NanoLiP) by Protein Block Copolymerization Approach

**DOI:** 10.3390/polym8060223

**Published:** 2016-06-06

**Authors:** Turgay Tay, Ender Köse, Rüstem Keçili, Rıdvan Say

**Affiliations:** 1 Department of Chemistry, Anadolu University, 26470 Eskisehir, Turkey; rsay@anadolu.edu.tr; 2 Karen Biotechnol Ltd., Anadolu University, Technol Pk, 26470 Eskisehir, Turkey; ender@karenbiotech.com; 3 Yunus Emre Vocational School, Department of Medical Services and Techniques, Anadolu University, 26470 Eskisehir, Turkey; rkecili@anadolu.edu.tr

**Keywords:** nanoenzyme, lignin peroxidase (LiP), photosensitive monomer, ANADOLUCA, tetramethylbenzidine (TMB)

## Abstract

This study describes the preparation of nanoprotein particles having lignin peroxidase (LiP) using a photosensitive microemulsion polymerization technique. The protein-based nano block polymer was synthesized by cross-linking of ligninase enzyme with ruthenium-based aminoacid monomers. This type polymerization process brought stability in different reaction conditions, reusability and functionality to the protein-based nano block polymer system when compared the traditional methods. After characterization of the prepared LiP copolymer nanoparticles, enzymatic activity studies of the nanoenzymes were carried out using tetramethylbenzidine (TMB) as the substrate. The parameters such as pH, temperature and initial enzyme concentration that affect the activity, were investigated by using prepared nanoLip particles and compared to free LiP. The reusability of the nano-LiP particles was also investigated and the obtained results showed that the nano-LiP particles exhibited admirable potential as a reusable catalyst.

## 1. Introduction

Lignin peroxidase (LiP, EC 1.11.1.14) is a heme-containing glycoprotein that has ability to catalyze H_2_O_2_ dependent-oxidative depolymerization of lignin [1,2]. One of the potential sources of LiP is *Phanerochaete chrysosporium (P. chrysosporium)*. It produces a group of isozymes, which have the ability to catalyze cleavage of C-C bond in lignin.

Immobilization of enzymes is commonly used in industry due to the various advantages, such as enhanced enzyme activity and stability in harsh operation conditions. The immobilization approaches, such as covalent binding [3,4], physical binding [5] and encapsulation [6,7,8,9,10,11], are widely used in enzyme immobilization. Among these immobilization techniques, adsorption is facile to conduct, but the binding of the enzyme molecules to the solid support is not strong compared to the covalent binding of enzymes. Adsorbed enzyme molecules are generally not stable enough. Thus, they cannot be used for long-term applications. On the other hand, covalent immobilization of enzymes results in an increase to enzyme stability. However, conformational restrictions may occur, and this leads to partial loss of enzyme activity.

Enzyme immobilization by chemical conjugation using artificial resins, such as polymers as solid supports, provides an increase in their thermal stability [12,13,14,15]. However, this method exhibits some drawbacks. The chemical conjugation does not always proceed in a straight manner and usually leads to significant loss of enzyme activity. The other approach for immobilization of enzymes and proteins is bioconjugation [16,17,18].

Protein-based artificial block copolymers have attracted significant attention from researchers [19]. These materials have two or more distinct blocks (protein and co-monomer/s), which are covalently bound each other. They also have considerable advantages, such as high stability and ordered protein 3-D conformations in the polymeric network, compared to traditional polymers. Protein-based artificial block copolymers can be prepared in the nano-scale, which provides a number of benefits for different applications in biotechnology, such as drug delivery, tissue engineering and *in vivo* imaging.

Enzyme and protein block corporation in nanostructures has brought a new perspective to protein based block copolymers with respect to unique functionalities and physical properties when compared to conventional block copolymers. These unique features of the protein-based block copolymers arise from the stable and ordered 3-D conformations of the incorporated protein blocks in the polymeric structure. The protein blocks have also ability to undergo self-assembly, which provides control of the formation of the protein-based polymeric structure in the micro- and nano-scale. The appropriate selection, positioning, and design of proteins in block copolymerization systems provide high levels of protein activities and utilities.

Micro emulsion polymerization (MP) is a widely used approach for the preparation of particles in the nano-scale. Large volumes of surfactant are consumed to prepare micro emulsion droplets and stabilize the obtained nanoparticles during the polymerization [20] with the MP technique. In addition, the type and volume of the surfactant used, and the pH effect the nanoparticle stability, are affected [21]. The cross-linking method, based on photosensitive Ru-amino acid monomers, called “ANADOLUCA” which is the abbreviation for amino acid monomer decorated and light underpinning conjugation approach, used in this study, is an efficient and facile approach. The nanoparticles carrying enzyme or protein molecules could be synthesized using this technique at room temperature. The considerable advantages of this method, compared to conventional immobilization methods, are the elimination of chemical activation of the support material using hazardous chemicals, such as cyanogen bromide, and ligand immobilization process.

In this work, we prepared nano-enzyme particles, with LiP, by using the photosensitive cross-linking polymerization approach, ANADOLUCA [22], which is used in the preparation of nanoparticles with narrow size distributions. The protein-based nano block polymer was synthesized by cross-linking of the ligninase enzyme with ruthenium-based amino acid monomers. This type polymerization process brought stability in different reaction conditions, reusability and functionality to the protein-based nano block polymer system when compared the traditional methods. The prepared nanoparticles were characterized by Zeta-Sizer. The activity studies of the LiP nanoparticles, by changing various and crucial factors, such as pH and temperature, were investigated using LiP nanoparticles. The obtained outcomes from these experiments were compared with free LiP. Reusability experiments for the prepared LiP nanoparticles were also performed in the last part of the study.

## 2. Experimental Section

### 2.1. Materials

Lignin peroxidase, 3,3′,5,5’ Tetramethyl benzidine (TMB), Triethylamine, Poly(vinyl alcohol) (PVA) (*M*_W_ 27,000), hydroquinone, methacryloyl chloride and all HPLC-grade solvents were supplied by Sigma-Aldrich (Steinheim, Germany).

### 2.2. Preparation of Nano LiP Particles

#### 2.2.1. Synthesis of Functional Monomer Metharyloyl Amidotyrosine (MATyr) and Photosensitive Ruthenium Based Aminoacid-Monomer bis(2-2’-bipyridyl)(MATyr)_2_-Ruthenium (II)

The functional monomer MATyr, which was used for the preparation of LiP nanoparticles, was synthesized according to a previously published procedure [23].

Bis(2-2’-bipyridyl)(MATyr)_2_-Ruthenium (II), based on the photosensitive cross-linking ANADOLUCA approach, was synthesized according to a previously published procedure [24].

**Figure 1:**
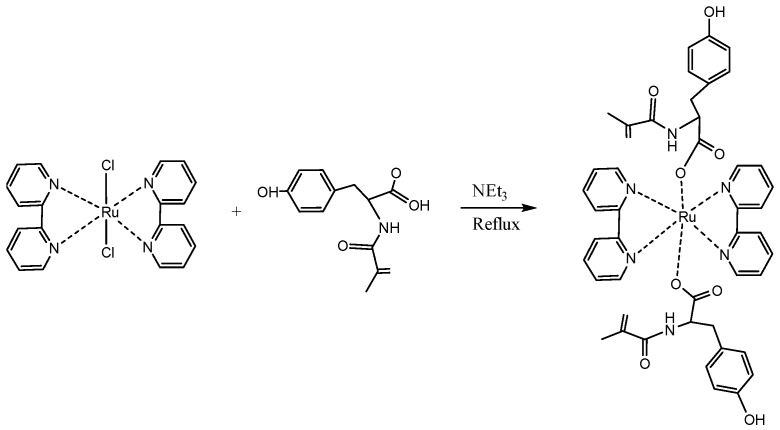
Schematic depiction of the preparation of bis(2-2’-bipyridyl)(MATyr)_2_-Ruthenium (II).

Schematic depiction of the preparation of bis (2-2’-bipyridyl)(MATyr)_2_-Ruthenium (II) is given in Figure 1.

#### 2.2.2. Preparation of Nano-LiP

**Figure 2:**
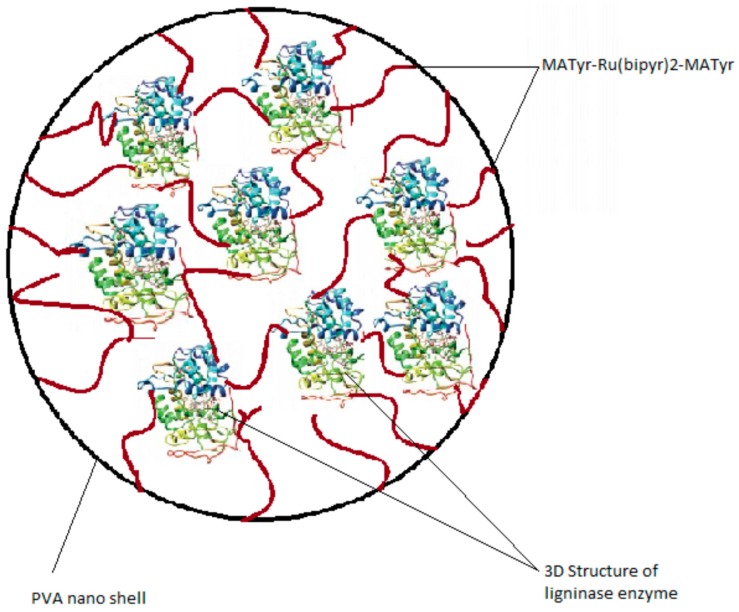
Schematic illustration of the synthesized nano lignin peroxidase enzyme.

LiP cross-linked nanoparticles (Nano-LiP) were prepared by using a previously reported procedure [25] based on microemulsion polymerization, as described in the following. It is schematically represented in Figure 2.

First, 0.5 g of PVA was dispersed in 45 mL of deionized water to prepare the microemulsion system. Then, 50 µL of bis(2-2’-bipyridyl)(MATyr)_2_-Ruthenium (II) was mixed with 1 mL of 5,000 ppm LiP and stirred for 1 h. Then, this solution was slowly added to 35 mL of PVA solution. Later, 20 mL of 0.02 g APS/50 mL H_2_O as initiator was added into the solution and mixed for 36 h under N_2_ atmosphere. Separation of the obtained LiP nanoparticles from the polymerization medium was performed by centrifugation at 14,000 rpm for 20 min. The nanoparticles were then washed with EtOH and deionized water to remove unreacted monomers and other compounds.

### 2.3. Characterization of Photosensitive Cross-Linked LiP Nanoparticles (Nano-LiP)

The average particle size and size distribution of the prepared LiP nanoparticles (NanoLiP) were determined by Zeta-Sizer (Malvern Instruments, Model 3000 HSA, Worcestershire, UK).

### 2.4. Activity Assay of the Nano-LiP

The enzymatic activity experiments of the LiP were carried out using a UV-Vis Spectrometer (Shimadzu UV 1800, Tokyo, Japan) using TMB as the substrate. In these experiments, 10 mM of TMB and 2 mM of H_2_O_2_ was prepared (substrate solution). One hundred microliters of LiP nanoparticles, dispersed in H_2_O, were added into 50 µL of this solution. The LiP activity assay was performed by measuring the absorbance at 650 nm using the following equation:

(1) $$U=\;\frac{∆A}{t\times \varepsilon_{650}\times l}\times V\times \frac{10^{6\;}\mu mol}{1\;mol}\times \frac{1\;L}{10^{6}\;\mu L}$$

where *U* represents the unit activity (µmol·min^−1^), ∆*A* is the absolute absorbance, ε is the molar extinction coefficient of TMB (M^−1^·cm^−1^), *V* is the total volume of solution (µL).

### 2.5. The Temperature and pH Effect on the Activity of Nano-LiP

Various temperatures, in the range of 25–70 °C, were applied to investigate the effect of temperature. Fifty microliters of nanoparticles were added into the 50 µL of 10 mM substrate solution and mixed for 30 min. The activity of NanoLiP was spectrophotometrically measured at 650 nm. To test the pH effect on the activity of nano-LiP, 100 µL of nanoparticles in H_2_O were mixed with 100 µL of 10 mM substrate solution at different pH values, varying 2 to 7. 50 mM Glycine-HCl (pH 2–3), 50 mM citrate (pH 4–5) and 50 mM phosphate (pH 6–7) buffers were used in these experiments.

After a 30 min incubation of substrate solution with the prepared LiP nanoparticles, the activity of Nano-LiP was calculated by measuring the absorbance at 650 nm.

## 3. Results and Discussion

### 3.1. Size Distribution Studies for Nano-LiP

**Figure 3:**
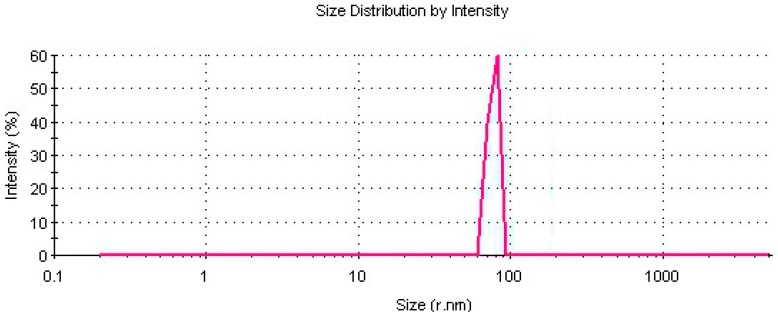
Size distrubition of the LiP nanoparticles.

The studies for the determination of average particle size of the prepared nano-LiP particles were done by using a Zeta-Sizer instrument. The average particle size of the nanoparticles was measured at about 90 nm, as shown in Figure 3.

### 3.2. The Temperature and pH Effect on the Activity of Nano-LiP

**Figure 4:**
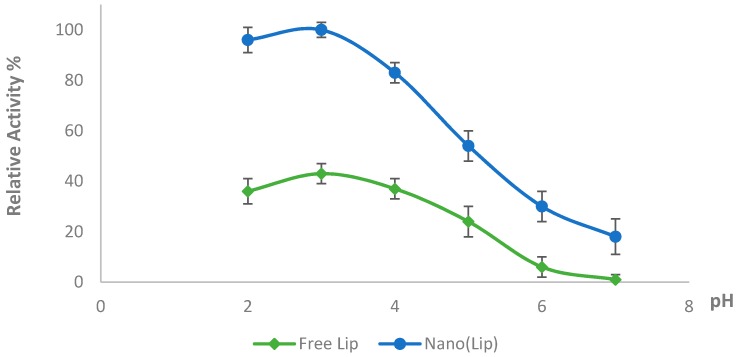
pH effect on the activity of free and Nano-LiP.

**Figure 5:**
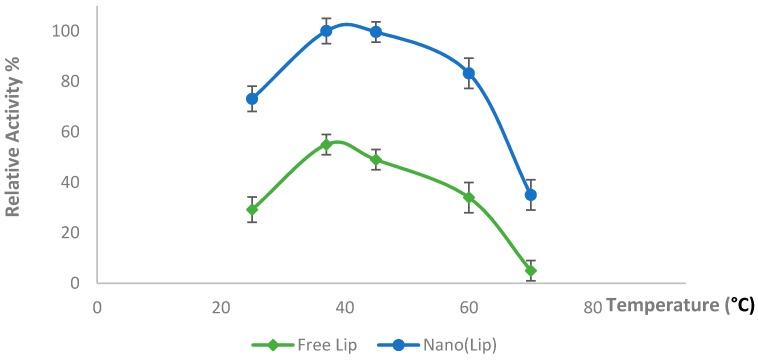
Temperature effect on the activity of free and Nano-LiP.

One of the most important parameters that affects the activity of enzymes is pH. In the current work, the optimum pH was determined at different pH values, varying from 2 to 7. The highest LiP activity was observed at 50 mM Glycine-HCl buffer, pH 3.0 (Figure 4). This result is consistent with other studies reported by different scientists [26,27]. Above a pH of 3, the LiP activity decreases, most probably due to the deprotonation of the groups that play crucial role for the catalysis in the active center of the enzyme. The temperature effects on the activity of the prepared Nano-LiP and free LiP were also investigated. Maximum activity was obtained at 40 °C, as can be seen in Figure 5.

In another study reported by Aster and Meunier [28], LiP was immobilized onto cyanogen bromide (CNBr) activated Sepharose-4B, Affi-Gel 15 and Affi-Gel 15 support materials. The maximum enzyme activity was obtained at pH 2.5, which is consistent with the results in our study. On the other hand, immobilized LiP activity significantly decreased after 40 °C, and the remaining relative activity was ~25% at 50 °C. In our study, Nano-LiP exhibited over 90% relative activity at 50 °C.

The prepared Nano-LiP displayed high relative activity (~80%) and thermostability at 60 °C. Asgher *et al.* [29] have prepared xerogels for immobilization of LiP. The LiP in immobilized form showed maximum activity at pH 4. The optimum temperature was found to be 40 °C. The relative activity was lower at higher temperatures, and ~70% relative LiP activity was obtained at 60 °C, which is lower than the results obtained in our study.

### 3.3. Reusability of Nano-LiP

**Figure 6:**
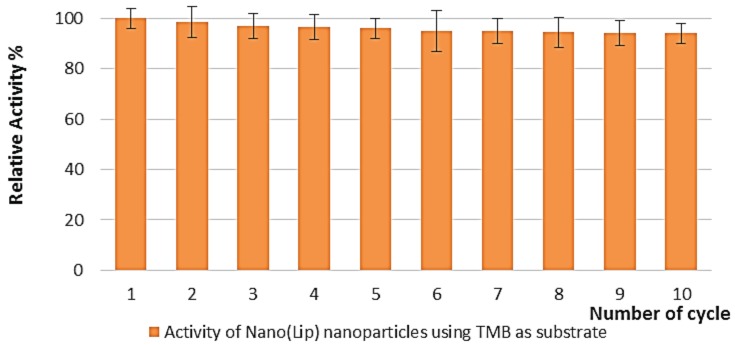
Reusability of the Nano-LiP particles.

The enzyme activity studies were repeated 10 times in order to determine the reusability of the prepared Nano-LiP. After each experiment, Nano-LiP particles were washed several times with distilled H_2_O and EtOH. The obtained results from these experiments showed that LiP nanoparticles are stable after 10 cycles (Figure 6).

Zungfang *et al.* prepared mesoporous silica particles for LiP immobilization [30]. In their study, 1,4-phenylene diisothiocyanate, cyanotic chloride, glutaraldehyde, and carbodiimide as activating agents were used for the covalent immobilization of LiP. The prepared immobilized LiP showed the highest activity at pH 3.5, and reusability studies showed that immobilized LiP starts to denaturate, and its enzymatic activity decreases, after the third cycle.

**Figure 7:**
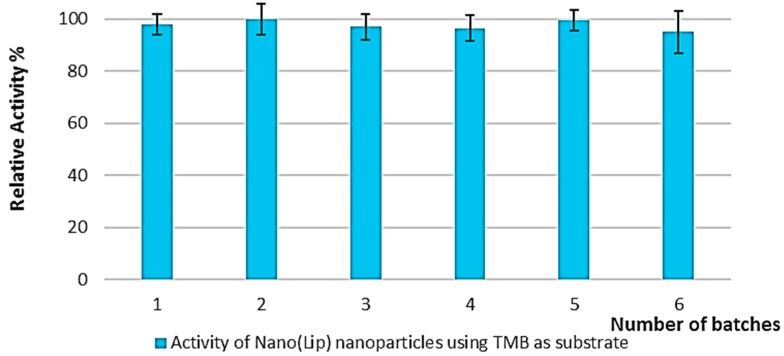
Activity of the nanoparticles prepared in different batches.

On the other hand, six different batches of the nanoparticles have also been prepared in the current study. The obtained results from these experiments showed that the different batches of the prepared nanoparticles displayed over 95% activity toward the substrate TMB (Figure 7).

### 3.4. Linearity and Repeatability Tests for Nano-LiP

**Figure 8:**
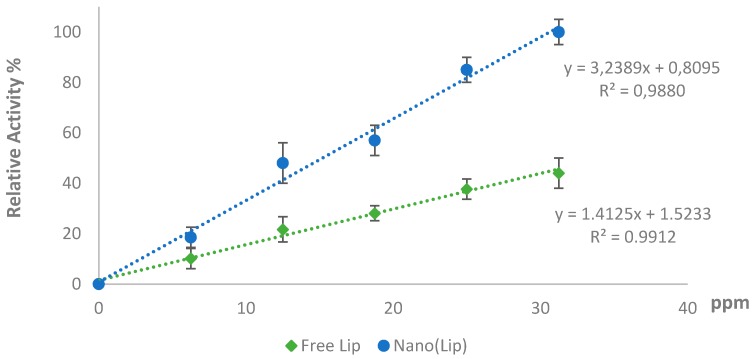
Linearity of free LiP and Nano-LiP.

Linearity of Nano-LiP and free LiP were also been studied. The results of these studies are shown in Figure 8. The obtained outcomes of these studies showed that LiP nanoparticles showed good linearity with regression coefficient of 0.9880.

**Figure 9:**
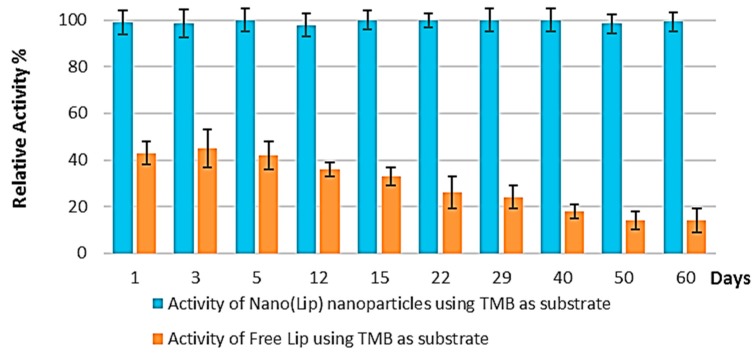
Repeatability of free LiP and Nano-LiP particles.

Experiments on the enzymatic activity of the prepared LiP nanoparticles were also performed on different days to investigate repeatability. The outcomes of the repeatability experiments are shown in Figure 9. The prepared Nano-LiP particles are quite stable for a long time and can be used several times without losing enzymatic activity toward the substrate TMB. The Nano-LiP particles retained ~100% activity for 60 days after storing at 4 °C, while the relative activity of the free LiP decreased to ~18%.

## 4. Conclusions

A new semi-synthetic (Nano-LiP) nanoenzyme LiP, which displays catalytic activity toward TMB, was prepared using photosensitive microemulsion capolymerization. The ligninase-based nano block polymer was synthesized by cross-linking of ligninase enzyme with ruthenium-based aminoacid monomers. The prepared Nano-LiP displayed high activity toward TMB at the optimum pH 3 at 40 °C. The Nano-LiP preserved their enzymatic activity with enhanced stability. The prepared LiP cross-linked copolymeric nanoparticles are stable even after 30 cycles of usage. This effective and straight-forward protein block copolymerization approach can be used for the preparation of reusable nano-enzymes in new enzyme engineering applications, and this process brought stability in different reaction conditions, and reusability and functionality to the protein-based nano block polymer system when compared with traditional methods.
